# Food Choices, Sustainability and Australian Native Foods: Perceptions among University Students

**DOI:** 10.3390/foods13111677

**Published:** 2024-05-27

**Authors:** Carla Vanessa Alves Lopes, Putu Novi Arfirsta Dharmayani, Rimante Ronto, John Hunter, Seema Mihrshahi

**Affiliations:** Department of Health Sciences, Faculty of Medicine Health and Human Sciences, Macquarie University, Macquarie Park, Sydney, NSW 2109, Australiaseema.mihrshahi@mq.edu.au (S.M.)

**Keywords:** sustainable diets, sustainability, native food, nutrition

## Abstract

(1) Background: Urgent changes in our food choices are needed for more sustainable, resilient and equitable food systems. Australian native plant-based foods have both environmental and health benefits. Including these foods in our diet may reduce both the risk of chronic diseases and the impact of climate change. This study explored the perceptions and attitudes towards food choices, sustainability and Australian native plant-based food among university students. (2) Methods: A cross-sectional online survey was completed by 212 university students in Australia from October to December 2022. Questions included information about sociodemographic characteristics, food choices, Australian native foods and the impact on sustainability. Logistic regression was used for analyses. (3) Results: Most participants recognised the impact of food choices on sustainability. There was a significant association between recognition of the impact of food choices on sustainability and the environmental and nutritional benefits of Australian native foods (OR = 2.89, 95% CI 1.29, 6.46, *p* = 0.010). Students who were familiar with or had tried Australian native plant-based foods were significantly more likely to recognise their environmental and nutritional benefits (*p* < 0.001). (4) Conclusions: Students who recognise the impact of food choices on sustainability and the benefits of Australian native foods are more likely to include native foods in their diet. More studies are needed to investigate the specific native foods consumed and the barriers and facilitators to the intake of these foods.

## 1. Introduction

The world has been facing a global syndemic, which refers to the occurrence of three pandemics at the same time and place—obesity, undernutrition and climate change [1,2]. According to the Lancet Commission report [1], the main syndemic drivers are land use, food and agriculture, transportation and urban design. At least 7 of the 17 Sustainable Development Goals are impacted by climate change and agriculture outcomes [3,4]. Food systems are significant emitters of carbon dioxide (CO_2_) and non-CO_2_ greenhouse gases (GHGs), contributing to 19–29% of GHGs global emissions [3,4]. Furthermore, the current global food systems are responsible for depleting non-renewable resources such as approximately 40% of global arable land [5] and about 70% of fresh water [5,6,7]. Extreme weather events can also impact access to food supplies and food prices, directly affecting food security [8]. It is estimated that for each 1 °C temperature anomaly, severe global food insecurity has increased by 1.4% [8]. In Australia, it is estimated that between 4% and 13% of the population is food insecure [9], with this number reaching more than 50% among university students [10]. Diet quality is also one of the major chronic diseases risks [11]. In 2018, dietary risks were responsible for 5.4% of the burden disease in Australia, including a diet high in red meat (0.9% of total burden), high in sodium (0.9%), low in legumes (1.2%) and low in whole grains (0.9%) [11]. All dietary risks combined contributed to 26% of stroke burden, 26% of bowel cancer, 26% of type 2 diabetes and 50% of coronary heart disease [11]. The 2022 Australian National Health Survey [12] showed that almost half of Australians (49.9%) had at least one chronic condition such as diabetes, heart disease or cancer.

Studies have shown that some changes in our food choices can positively impact our health and the environment. Increasing the consumption of fruits, vegetables and whole grains and reducing the consumption of red meat and ultra-processed foods can prevent chronic diseases and have lower environmental impacts [13,14]. Traditional, native and local foods also play a significant role in improving human health and mitigating climate change. Traditional foods are defined as being edible species belonging to a local and native environment that are culturally acceptable [15,16]. Native foods are those that have been part of the natural landscape for centuries, are adapted to the local climate and soil conditions and have been part of the Indigenous diet of a particular region or ecosystem [17]. Local foods refer to foods that are produced, processed and consumed within a short distance of where they are grown, usually connecting farmers and consumers [18], and can include native and non-native species. Studies have shown that including traditional and native foods in our current system and diet can enhance food diversity, an important strategy to address diet-linked chronic diseases [19,20]. Our recent review has shown the social, economic and environmental benefits of Australian native plant-based foods for more sustainable food systems [21]. Several studies have identified the health and nutritional benefits of these plants, including anticancer, antidiabetic and anti-inflammatory activities; their cultural meaning for Aboriginal people; and also some environmental benefits such as promoting biodiversity, carbon sequestration and pollination and being salinity, drought and heat tolerant [21]. Likewise, in Africa [22] and America [23,24,25], studies have also found the importance of native foods for food diversification and their environmental and health benefits to combat diet-linked chronic diseases, such as diabetes and cancer. 

Some notable examples of Australian native foods associated with antidiabetic activities are wattle seeds (*Acacia aneura*) and *Acacia coriacea* [26,27,28]. Some flakes and crackers developed with Australian native foods also showed a low glycaemic index [29]. Many Australian plant-based foods are richer in protein than non-native species, such as native rice [30], Pindan walnut [31] and *Acacia* seeds [32,33,34,35] and others are rich in micronutrients, such as *Eucalyptus pachyphylla F. Muell* (*Myrtaceae*) seeds—one of the most magnesium-rich foods (501 mg/100 g) [36]—and Kakadu plum—the highest source of vitamin C in the world [37,38,39]. 

In addition to the direct health and ecosystem benefits, Australian native plants are part of Aboriginal cultural and spiritual connection and respect for natural resources [40,41,42,43], which are principles and values that can also have environmental benefits. Some Aboriginal food practices linked to Australian native plants also have positive environmental impacts, such as seasonal-based food consumption [44] and ecological knowledge, including the right time to plant, harvest and burn [40,41,44,45]. There is enough support from the literature towards an increase in plant-based foods in our diets, including fruits, vegetables and Australian native species. 

Although the evidence shows the impact of food choices on human health and the environment, only 5.4% of adults in Australia met the fruit and vegetable recommendation in 2017–2018 (two or more servings for women and five or more servings for men) [46]. Many social determinants can impact eating habits, such as a lack of access and money constrains [47], cultural determinants [48,49] and food and nutritional knowledge [50,51,52,53]. Studies have shown the importance of understanding the impacts of food choices on the environment and more sustainable eating habits [54,55,56]. A recent systematic literature review also showed the positive effects of web-based nutrition education interventions on knowledge and attitudes towards sustainable and healthy diets in young adults [57]. Nutritional education interventions in some traditional communities, including gardening and cooking workshops with native foods, have also shown the potential to improve knowledge about these foods and increase their consumption [58]. However, there is a gap in studies on knowledge and consumption of native foods in the general population, as these studies have been targeting First Nations People. This study aims to explore the perceptions and attitudes towards food choices, sustainability and Australian native plant-based foods among university students. Our hypothesis is that (1) university students who recognise the impact of food choices on sustainability and the environmental and nutritional benefits of Australian native plant-based foods are more likely to incorporate these foods into their diet and (2) university students who are familiar with Australian native foods are more likely to recognise the impact of food choices on sustainability.

To support our hypothesis, we have used the behaviour change wheel (BCW) framework [59], which provides a comprehensive method for designing behaviour change interventions. This framework supports that behaviour change depends on individual’s capability (C), opportunity (O) and motivation (M). The framework also identifies nine intervention fields (education, persuasion, coercion, incentivisation, training, restriction, environmental restructuring, modelling and enablement) and seven policy categories (guidelines, regulation, legislation, communication, environmental/social planning and service provision) for designing interventions for behaviour change [59]. In this exploratory study, we assessed students’ knowledge about the impact of food choices on sustainability and the environmental and nutritional benefits of Australian native plant-based foods (capability), their attitudes (motivations) towards these foods and their previous exposure (opportunity) to these foods. We also investigated the general fruit and vegetable consumption along with their barriers. The preliminary results from this study will be used as a guide to design interventions to promote sustainable diets and include intake of Australian native foods. 

Research Questions:

- What are university students’ perceptions and attitudes towards the impact of their food choices on sustainability?
- Do the university students meet the guidelines for fruit and vegetable consumption? What are their barriers?
- How familiar are university students with Australian native plant-based foods?
- Is there any association between recognising the benefits of Australian native foods and the chance of including these foods in their diet?

## 2. Materials and Methods

### 2.1. Design and Participant Recruitment

This cross-sectional study is part of the project “Co-designing an intervention to improve food security and healthy eating among university students”, which was approved by the Macquarie University Human Research Ethics Committee (Project ID: 11860; Approval number 520221186039979). A survey was conducted using an online platform, Research Electronic Data Capture (REDCap), between October 2022 and December 2022. The inclusion criteria were being a student enrolled at Macquarie University and aged at least 18 years. Convenience sampling was employed to recruit the participants. The survey link and a quick response (QR) code were advertised using flyers on campus, the university newsletter and social media. In addition to online recruitment, an intercept survey was also conducted by approaching the students on campus, where they could scan the QR code and complete the survey immediately or at their convenience. A Digital Participant Information and Consent Form was completed before commencing the survey. Participants were assured that participation in the study was voluntary and anonymous. 

### 2.2. Measures

#### 2.2.1. Perceptions of Food Choices and Australian Native Foods and Their Impact on Sustainability

The survey included two statements and one question related to the topic. The statements included were: “My food choices can impact the environment” and “Some Australian native plant-based foods are rich in nutrients and have less environmental impact”. For the statements, the participants responded on a 5-point Likert-type scale—strongly agree, agree, not sure, disagree and strongly disagree. For the question, “Have you ever heard about Australian native foods? Have you ever tried any Australian native food?” the participants responded with one of the following answers: “Yes, I know some Australian native foods, and I have tried some of them”, “I have heard about Australian native foods, but I have never tried any” and “No, I have never heard about Australian native foods, but I am interested to know more about it”.

Given the exploratory nature of this study, these questions were designed to capture initial insights into the university students’ perceptions and attitudes regarding sustainable food choices and Australian native plant-based foods. The questions were not formally validated, as they were developed based on the objectives of the study. 

#### 2.2.2. Reported Behaviours and Perceptions of Fruit and Vegetable Consumption

Validated questions from the Australian National Health Survey 2017–2018 [46] were used to measure fruit and vegetable consumption. The answers were based on the number of servings per day, ranging from less than one serving to six or more, or “I don’t eat fruit/vegetables”. The data were recoded to <2, ≥2 and <5 and ≥5 for fruits and vegetables to facilitate the analysis based on the Australian Dietary Guidelines [60]. The New South Wales Population Health Survey questions were used to explore behaviours and perceptions of fruit and vegetable consumption adequacy, barriers to increasing fruit and vegetable intake and perceived access to fruit and vegetables [61,62,63].

#### 2.2.3. Sociodemographic

Sociodemographic characteristics included age, gender, country of birth, Indigenous status, student status (domestic or international) and their current degree (PhD, postgraduate, undergraduate or other).

### 2.3. Data Analysis

Statistical analyses were performed, including students who completed the survey (n = 212), using STATA/MP version 18 (StataCorp, LP, College Station, TX, USA). Descriptive analysis was performed for all continuous variables presented using mean and standard deviation (SD), whereas the categorical variables were described using frequency (n) and percentage (%). Univariate logistic regression was performed to analyse the association between the recognition of the environmental and nutritional benefits of Australian native foods and the recognition of the impact of food choices on sustainability and Australian native food intake. With respect to the question about Australian native foods, the multivariable logistic regression was adjusted for age and student status (i.e., domestic and international student). The significance level was set at *p* < 0.05.

## 3. Results

A total of 320 students signed the consent form and started responding to the survey. However, 108 respondents were excluded for the following reasons: starting but not completing the survey questionnaire (n = 107) and being under the age of 18 years (n = 1).

### 3.1. Sociodemographic Characteristics

Table 1 shows the characteristics of the study participants. The participants were predominantly female (72.64%), the average age was 24.1 ± 6.9 years, 67.92% were domestic students, most (61.32%) were undergraduate students and enrolled in the Faculty of Medicine (35.85%) and Faculty of Arts (34.43%) and less than 1% were Indigenous students. 

There was no significant difference between sociodemographic groups, comparing the percentages of responses to recognition of the environmental and nutritional benefits of Australian native foods, recognition of the impact of food choices on sustainability and Australian native food intake. 

### 3.2. Reported Behaviours and Perceptions of Fruit and Vegetable Consumption

Table 2 shows that 45.28% of the participants met the daily recommendation for fruits (at least two servings using the Australian Dietary Guidelines), and only 5.66% met the recommendation for vegetables (five servings or more). Although most participants did not meet the fruit and vegetable recommendations, 51.42% and 42.92% perceived their fruit and vegetable consumption as adequate, respectively. The main barriers to consuming more vegetables were the perception that they already had enough in their diet (19.81%), the high price of vegetables (18.87%) and habit (18.40%). For fruit consumption, the main barriers were the high price of fruits (27.36%) and habit (26.89%). Most students perceived easy access to fruit (67.92%) and vegetables (72.64%).

### 3.3. Perceptions of Food Choices, Australian Native Foods and Sustainability

Most participants (84.43%) recognised (strongly agreed or agreed) the impact of food choices on sustainability (Table 3). The nutritional and environmental benefits of Australian native plant-based foods were familiar to more than half of the participants (52.83%). However, 46.70% of the participants were not sure about this information, and less than 1% (0.47%) disagreed with this statement. Finally, 42.45% stated that they had never heard about Australian native foods, 27.83% had heard about these foods but had never tried any and 29.72% of the participants had tried some Australian native foods.

### 3.4. Association between Food Choices, Australian Native Foods and Sustainability

Table 4 shows the results of univariate logistic regression between the recognition of the benefits of Australian native foods, the recognition of the impact of food choices on sustainability and the intake of Australian native food. A significant association was found between the recognition of the impact of food choices on sustainability and the environmental and nutritional benefits of Australian native foods (OR = 2.89, 95% CI 1.29, 6.46, *p* = 0.010). The adjusted model confirmed a significant association between those who had heard (OR = 3.72, 95% CI 1.84, 7.51, *p* = 0.001) or tried (OR = 16.42, 95% CI 6.96, 38.74, *p* = 0.001) Australian native foods and those who were familiar with the nutritional and environmental benefits of these foods. These results show that those who had tried the Australian native foods are more than 16 times more likely to recognise the nutritional and environmental benefits of Australian native foods. No significant statistical association was found between the recognition of the impact of food choices on sustainability and fruit and vegetable consumption. 

No significant statistical association was found between the recognition of the impact of food choices on sustainability and the Australian native food intake (Table 5).

## 4. Discussion

To our knowledge, this is the first study to explore the perceptions and attitudes towards food choices, sustainability and Australian native plant-based foods. Overall, the results indicated that although the participants recognised the impact of food choices on the environment and the benefits of Australia native foods, a greater effort is needed to transfer this awareness into sustainable food practices. Approximately half of the students reported meeting the daily recommendation for fruits, and less than 6% met the recommendation for vegetables. However, about half of the participants perceived their fruit and vegetable consumption as adequate. Almost 85% of the participants recognised the impact of food choices on the environment. More than 52% recognised the nutritional and environmental benefits of Australian native plant-based foods, yet less than 30 had tried some Australian native foods. 

In the present study, there is no significant difference in fruit and vegetable consumption based on sociodemographic characteristics, nor were there any differences in recognition of the impact of food choices on sustainability, the environmental and nutritional benefits of Australian native foods and recognition of the Australian native food intake between sociodemographic groups. 

Many other studies also identified low fruit and vegetable consumption among young adults in Australia [61,64] and other countries [65]. Young adults in Australia are one of the populations with the lowest vegetable intake compared to other group ages [66,67], and it is important to study the reasons for this in order to design interventions to improve it. 

Although the Australian and international guidelines show fruit and vegetable consumption as part of a sustainable diet [13,14,60,68], and most of the participants (85%) in our study recognised the impact of food choices on the environment, they did not meet these recommendations. Individual and socio-environmental factors may influence fruit and vegetable intake among young adults, such as taste preferences, perceived time barriers to healthy eating, home availability of fruits and vegetables, home availability of unhealthy foods and home food preparation [69]. In our study, affordability, a gap in knowledge about the recommended daily servings of fruit and vegetable, and a gap in skills to prepare (mainly related to less time to prepare and more interesting and tasty ways to serve) fruits and vegetables were the main reported barriers to increasing fruit and vegetable consumption. However, accessibility was not a main issue for most students, as 72.64% perceived easy access to vegetables and 67.92% perceived easy access to fruits. 

A scoping review, including seventy-one papers worldwide, assessed the factors associated with increased vegetable consumption among college students [70]. Some of the main factors presented were living in the family home, having a normal weight, greater perception of happiness and less stress and pressure, higher socioeconomic status, the importance of healthy eating, having breakfast, early mid-point of sleep and nutrition knowledge [70]. Some studies reported the association between cooking skills and greater vegetables and fruit consumption and improvements in healthier eating behaviours [71,72]. However, a quasi-experimental controlled study with healthy Australian adults showed that a seven-week food literacy cooking program had no significant differences in intake of fruit and vegetables between the intervention and control group [73].

Previous studies have also shown that availability and affordability are two predominant factors that interfere with food security and choices [74,75,76]. Miller et al. [77] assessed the availability, affordability and consumption of fruits and vegetables in 18 countries across income levels. The study showed that increased costs of fruits and vegetables were associated with reduced consumption [77]. An online survey of the Australian adults also showed that almost 40% of participants reported that cost was a barrier to buying fruits and vegetables [78].

Similar findings to our study have been shown in an online survey with adults in New South Wales, Australia. In this study, it was reported that the perceived adequacy of fruit and vegetable consumption can be a barrier to increasing consumption [62]. The study also showed a disconnect between meeting the guidelines and perceived adequacy of consumption, with 53.7% of those participants who perceived adequacy of vegetable consumption and 21.3% of those who perceived adequacy of fruit consumption not meeting the guidelines. 

Our study supports that the majority of young adults were aware of the impact of food choices on the environment. Another study in Australia also explored the perspectives of young Australians about sustainable and healthy diets and found that most participants were familiar with some aspects of sustainable and healthy diets [79]. Work done by Gonzalez et al. [80] on the Spanish adult population reported that more than 50% of the participants recognised the relationship between food sustainability and environmental impacts and local foods, but they were less familiar with terms such as carbon footprint. Another study in Spain with a university population also found that most participants reported they had heard about the environmental impact of food or the Sustainable Development Goals, but they presented lower level of knowledge about specific aspects of sustainability, such as greenhouse emissions, carbon footprint and biodiversity [81]. A study conducted in a Turkish university showed that around 60% of the students had heard about sustainable nutrition, but understanding was lacking, and the majority of male participants (71.2%) agreed that foods have no environmental impacts [82]. Nonetheless, many of them may lack sufficient knowledge of native foods and locally produced foods, contributing to the gap between their perceived impact on the environment through food choices and the actual consumption of native foods in the general population.

Another important finding in our study is that there is a significant association between being familiar with the nutritional and environmental benefits of Australian native foods and having the chance to try these foods, highlighting that nutritional and sustainability knowledge is an essential factor that may impact food choices. Aureli and Rossi [83] evaluated the association between nutritional knowledge and adherence to the Mediterranean diet in Italy. The authors showed that the participants with the highest level of nutritional knowledge were more likely to adhere to the Mediterranean diet. However, a systematic review that examined the relationship between nutrition knowledge and dietary intake in adults showed a positive but weak association between higher nutritional knowledge and dietary intake [84]. A cross-sectional study in Spain with students and professionals in health sciences found a significant positive correlation (*p* < 0.001) between deeper knowledge and attitudes towards sustainable foods and higher consumption of vegetables and fruits [54]. 

Studies on First Nations Communities have associated the importance of nutritional and environmental knowledge of traditional foods and their impact on food choices. A traditional food program in Inuvik, Western Canadian Artic, showed that promoting youth engagement with traditional foods, including improving traditional knowledge, increased access to traditional foods and is a potential way to improve food security [85]. An exploratory study in remote Aboriginal communities in Australia reported that traditional food is consumed frequently by these community members (at least fortnightly), and 40% of those who reported being food insecure consumed traditional foods during these times [86]. An intervention to promote healthy eating among American Indian children included incorporating traditional growing practices and the preparation of traditional foods into the school curriculum, showing that it may be a potential strategy for increasing fruit and vegetable consumption [87]. In India, an intervention with traditional foods for rural Dalit mothers and young children also showed that traditional foods increased mothers’ energy, protein and iron intake [88].

Strategies such as these in Indigenous communities can be used to expand and promote Australian native foods among students. The behaviour change wheel framework [59] can be utilised to design interventions targeting capability, opportunity and motivation. Educational workshops can enhance students’ capability by increasing their knowledge about the benefits of native foods. Creating opportunities for students to access these foods on campus and in the general market through partnerships with local and Aboriginal suppliers and incorporating native foods into campus dining services can facilitate consumption. Motivational strategies, such as social media campaigns and university events and activities, can influence students’ attitudes and preferences. Other hands-on experiences can include gardening, cooking workshops and tasting sessions to engage students and encourage the inclusion of native foods in their diets. Importantly, all these efforts must be guided by policies and led by Aboriginal people to ensure their self-determination and sovereignty, recognising their cultural and historical connection to these foods. 

### Strengths and Limitations

This study has some important strengths and limitations that should be noted. Although the evidence shows several health, nutritional, cultural, economic and environmental benefits of Australian native foods [21], this is the first study to explore the perceptions of Australian native foods and sustainability among the general (non-Indigenous) population. Although information about the perception of food choices, Australian native foods and their impacts on the environment was collected, further details were not captured. Further research is needed to collect specific knowledge and perceptions about sustainability in food systems such as food waste, local and seasonal fruit and vegetable consumption, carbon footprint, greenhouse gas emissions, food groups and their impacts on the environment. Also, the definition of native food was not provided and, the specific names of Australian native foods the participants had tried were not collected, and so there may have been a bias in our research. The two subjects (nutritional and environmental benefits) considered in the native food statement do not allow us to know which part of the statement the participants answered. The lack of information of the consumption of all food groups to discuss their impacts on the environment and correlate it with sustainability knowledge is also a gap in our study. We also acknowledge that the lack of formal validation for the primary questions is a limitation of our study. However, the primary goal was to gather preliminary information that could inform future research. Further research using validated tools is necessary to investigate the types and amounts of native foods consumed, including frequency data. It is also important to note that this is an exploratory study with a small sample size collected at a single university, which is a limitation for the generalisability of the results to the wider university population. Our study did not present the current availability of Australian native foods on the market. Further research should aim to provide detailed data on the commercial availability of these foods, which may be one of the barriers for their consumption. 

## 5. Conclusions

Our findings suggest that university students in this particular sample are familiar with the impacts of food choices on the environment, and some are familiar with the nutritional and environmental benefits of Australian native foods. However, attitudes towards sustainable diets, such as fruit and vegetable consumption and Australian native food consumption, should be improved. Nevertheless, due to the limitations of our sample size, we are not allowed to generalise these results to the broader population of university students in Australia. 

Increasing population knowledge about sustainable diets and the health and environmental benefits of some food groups and native foods may be a positive influence on increasing sustainable food consumption. Our preliminary findings show the importance of integrating sustainability education into university curricula to improve knowledge and change behaviours towards sustainable food choices, including native foods. The similarities with previous studies reinforce the persistent barriers to healthy eating, such as affordability, which need to be addressed through community-based interventions and policies. 

The findings of this study can inform further research among the Australian population, including more details about food consumption and native foods. Also, these results can support some interventions among university students to improve their diets to include more sustainable options. Further research is needed to assess the knowledge and consumption of Australian native foods among Australian population, as well as their barriers and facilitators. This study may be a first step to inform future efforts to promote more sustainable diets including native foods among university students in Australia. 

## Figures and Tables

**Table 1:** Participant sociodemographic characteristics (n = 212).

Variable	n	%
Age (M ± SD)	24.1 (6.9)	--
Age group		
18–25 years old	149	70.28
26–30 years old	36	16.98
>30 years old	27	12.74
Gender		
Female	154	72.64
Male	54	25.47
Other	3	1.42
Prefer not to say	1	0.47
Indigenous status		
Yes	2	0.94
No	210	99.06
Country of birth		
Australia	105	49.53
Other countries	107	50.47
Student status		
Domestic	144	67.92
International	68	32.08
Degree		
PhD	15	7.08
Postgraduate	65	30.66
Undergraduate	130	61.32
Other	2	0.94
Faculty		
Faculty Medicine, Health and Human Sciences	76	35.85
Faculty of Arts	73	34.43
Faculty of Science and Engineering	31	14.62
Macquarie Business School	30	14.15
Macquarie University International College	2	0.94

Note: M = mean; SD = standard deviation.

**Table 2:** Reported behaviours and perceptions related to fruit and vegetable consumption (n = 212).

Variable	n	%
Fruit consumption ^a^		
<2 servings	116	54.72
≥2 servings	96	45.28
Vegetable consumption ^a^		
<5 servings	200	94.34
≥5 servings	12	5.66
Perceived adequacy of fruit consumption		
Too little/do not know	121	57.08
About right/too much	91	42.92
Perceived adequacy of vegetable consumption		
Too little/do not know	103	48.58
About right/too much	109	51.42
Barrier to eating more fruit		
Habit	57	26.89
Too expensive	58	27.36
Already have enough in my diet	41	19.34
It takes too long to prepare	10	4.72
Not enough interesting and tasty ways to serve	17	8.02
Limited access to good quality fruit	7	3.30
Other	22	10.38
Barrier to eating more vegetables		
Habit	39	18.40
Too expensive	40	18.87
Already have enough in my diet	42	19.81
Don’t know how to prepare	12	5.66
It takes too long to prepare	39	18.40
Not enough interesting and tasty ways to serve	29	13.68
Limited access to good quality vegetables	7	3.30
Other	4	1.89
Perceived easy access to fruit		
Strongly disagree/disagree	35	16.51
Not sure	33	15.57
Strongly agree/agree	144	67.92
Perceived easy access to vegetable		
Strongly disagree/disagree	29	13.68
Not sure	29	13.68
Strongly agree/agree	154	72.64

^a^ Following the Australian Dietary Guidelines.

**Table 3:** Recognition of the impact of food choices on sustainability and recognition of the environmental and nutritional benefits of Australian native foods (n = 212).

Variable	n	%
Food choices can impact the environment		
Strongly agree	95	44.81
Agree	84	39.62
Not sure	21	9.91
Disagree	9	4.25
Strongly disagree	3	1.42
Some Australian native plant-based foods are nutrient rich and have less environmental impact		
Strongly agree	40	18.87
Agree	72	33.96
Not sure	99	46.70
Disagree	1	0.47
Strongly disagree	0	0
Heard about or tried Australian native foods		
No, I have never heard about Australian native foods	90	42.45
I have heard about Australian native foods, but I have never tried any	59	27.83
Yes, I know some Australian native foods and I have tried some of them	63	29.72

**Table 4:** Association recognition of the environmental and nutritional benefits of Australian native foods with recognition of the impact of food choices on sustainability and Australian native food intake (n = 212).

Independent Variables	Unadjusted			Adjusted †		
	OR	95% CI	*p*-Value	OR	95% CI	*p*-Value
Food choices can impact the environment						
Yes ^a^	3.05	1.37–6.78	0.006 **	2.89	1.29–6.46	0.010 **
No ^b^	1	-	-	1	-	-
Heard of/tried Australian native foods						
No, I have never heard about Australian native foods.	1	-	-	1	-	-
I have heard about Australian native foods, but I have never tried any.	3.74	1.86–7.50	<0.001 ***	3.72	1.84–7.51	<0.001 ***
Yes, I know some Australian native foods and I have tried some of them.	16.5	7.08–38.46	<0.001 ***	16.42	6.96–38.74	<0.001 ***

OR: odds ratio; CI: confidence interval; † Adjusted for age and student status (i.e., domestic and international student); ^a^ strongly agree/agree; ^b^ strongly disagree/disagree/not sure; ** indicates a significant association (*p* ≤ 0.01); *** indicates a significant association (*p* < 0.001). Dependent variable: recognition of the environmental and nutritional benefits of Australian native foods.

**Table 5:** Association between recognition of the impact of food choices on sustainability and the Australian native food intake (n = 212).

Independent Variables	Unadjusted		
	OR	95% CI	*p*-Value
Heard of/tried Australian native foods			
No, I have never heard about Australian native foods.	1	-	-
I have heard about Australian native foods, but I have never tried any.	1.38	0.55–3.46	0.494
Yes, I know some Australian native foods and I have tried some of them.	1.30	0.53–3.16	0.566

OR: odds ratio; CI: confidence interval. Dependent variable: recognition of the impact of food choices on sustainability.

## Data Availability

The original contributions presented in the study are included in the article, further inquiries can be directed to the corresponding author.
